# Report of a Meeting: An Expert Consultation on Body Composition and Adiposity for Children and Adolescents in All Their Diversity

**DOI:** 10.1016/j.cdnut.2025.107475

**Published:** 2025-06-03

**Authors:** Lauren E O’Connor, Lucero Lopez-Perez, Ricardo X Martinez, Maureen K Spill, Juan Pablo Peña-Rosas, Amanda J MacFarlane

**Affiliations:** 1 Texas A&M Agriculture, Food, and Nutrition Evidence Center, Fort Worth, TX, United States; 2 Nutrition and Food Safety, Division of Healthier Populations, World Health Organization, Geneva, Switzerland; 3 Nutrition Research Division, Health Canada, Ottawa, ON, Canada

**Keywords:** obesity, overweight, body composition, diagnostics, anthropometry, children, adolescents

## Abstract

We convened experts to discuss methods for measuring body composition and diagnosing obesity among infants, children, and adolescents aged 0–19 y. The motivation for this meeting was to inform critical decisions for a systematic review protocol to assess the diagnostic test accuracy (DTA) of body mass index (BMI)-for-age and sex for diagnosing infants, children, and adolescents with excessive adiposity that can impair health. Thirty-nine clinicians and researchers from 23 countries provided written responses and/or attended 1 of 2 virtual meetings held in January 2024. Experts were asked to share their perspectives about methods and clinical tests used to measure body composition, including thresholds and adiposity types (i.e., total or central), for diagnosing obesity for infants, children, and adolescents. Experts suggested that deuterium oxide dilution, magnetic resonance imaging, dual energy X-ray absorptiometry, air displacement plethysmography, hydrostatic weighing, and multicompartmental models were acceptable to measure body composition, with the 4-compartmental model preferred. Waist circumference and bioelectrical impedance were preferred clinical tests to use either alone or in combination with BMI for diagnosing obesity; preferential use was country specific. Most experts preferred fat mass index (fat mass/height^2^) to % body fat as the metric, because it is more sensitive to changes over time and depends on age and height, similar to BMI. Experts agreed that total and regional adiposity are important for determining metabolic risk related to obesity, but using central adiposity for diagnosing obesity is challenging due to variations in body type. All agreed that age, race, ethnicity, and puberty stage should be considered when defining thresholds of obesity. This input from experts informed the systematic review protocol for an assessment of the DTA of BMI to support the World Health Organization’s guideline development for the integrated management of children and adolescents with obesity in all their diversity.

## Introduction

The World Health Organization (WHO) is developing living guidelines for the integrated management of children and adolescents with obesity. These guidelines will provide a primary health care approach to improve health, functioning, and reduce obesity-associated disability for those aged 0–19 y in all their diversity, inclusive of any gender, age, disability status, sexual orientation, cultural and socioeconomic background, and other characteristics. These guidelines will *1*) provide recommendations on individual, family, community, or home-based interventions, *2*) outline science-informed strategies to support well-being, and promote healthier weight achievement and maintenance, and *3*) provide guidance on diagnosis, monitoring, treatment, and follow-up for children and adolescents with obesity [1,2]. Over 35 systematic reviews (SRs) are being conducted on a breadth of topics, including the challenges of defining and diagnosing obesity in this age group, to inform the WHO’s guideline development process.

The current recommended method used to diagnose obesity in infants, children, and adolescents is BMI (in kg/m^2^) specific to age and sex [[3], [4], [5], [6], [7], [8]]. However, there is debate as to whether and how BMI-for-age and sex should be used for this purpose. BMI-for-age and sex tends to have moderate sensitivity and high specificity for diagnosing obesity in infant, children, and adolescent populations [[9], [10], [11], [12]]. Moderate sensitivity results in ∼30% of missed diagnoses, which can delay necessary treatment to prevent long-termmetabolic, physical, and mental health consequences [13]. BMI-for-age and sex poses particular challenges for those under 6 y of age [14,15] as well as children of Asian, Indian, or African descent due to differences in muscle development, body fat distribution, and puberty onset [16,17]. A diagnosis of obesity, independent of accuracy or whether health risks are present or not, can lead to stigmatization, mental health concerns, disordered eating, and body image issues for children and adolescents [18,19].

Research is needed to assess whether a diagnostic tool other than BMI, or one used in combination with BMI, can improve the accuracy of obesity diagnoses for infants, children, and adolescents. To support the WHO guideline development process, the Texas A&M Agriculture, Food, and Nutrition Evidence Center will conduct an SR to assess the diagnostic test accuracy (DTA) of BMI-for-age and sex for screening or diagnosis of obesity in children and adolescents aged 0–19 y in all their diversity. A series of consultations were held with international experts on childhood obesity, human growth and development, and body composition to solicit feedback and inform development of the DTA SR protocol. Therefore, the objective of this report was to present a narrative summary of compiled feedback from the expert consultation meetings that were used to inform methodological decisions for the DTA SR protocol (https://osf.io/vzwjt). We also present a brief background on the DTA framework and history of the main diagnostic test of interest (BMI-for-age and sex).

## DTA Framework

As described in the *Cochrane Handbook for Systematic Reviews of Diagnostic Test Accuracy* [20], the objective of a DTA SR is to summarize available research that estimates the ability of an *index test* (i.e., a test under investigation) to correctly identify individuals who have or do not have the target condition or disease. Diagnostic accuracy is assessed by comparing the performance of an index test(s) to the performance of a reference standard. Cochrane defines a reference standard as a highly reliable and widely accepted test to which the accuracy of the index test is compared. Accuracy is generally quantified by sensitivity, specificity, predictive values, area under the receiver operating characteristic curve, and likelihood ratios. The accuracy of several index tests can also be compared with each other using 1 or more reference standards in a comparative DTA review.

## BMI-for-Age and Sex

Adolphe Quetelet noted in 1842 that body weight in humans increases proportionally to height^2^ [21]. Referred to as Quetelet’s index for over 100 y, Ancel Keys termed weight/height^2^ as "body mass index," or BMI, in 1972 [22]. However, weight is not proportional to height^2^ during childhood and adolescence. The power of height may vary from 2.5 to 3.5 at various stages of development because body fat amount and distribution fluctuate during this period of maturation [23,24]. The use of BMI *z*-scores for children was proposed in 1982 after a study suggested that BMI was the superior metric compared with different weight-for-height indexes, such as weight/height^p^ or weight/height^3^. The study suggested that weight was independent from height, as was assumed for adults prior, and that there were associations between BMI and adiposity assessed via skinfolds for any age and sex [25,26]. Although this is just a brief history of the evolution of the use of BMI to assess adiposity in children and adolescents, previously published articles provide an in-depth history and mathematical overview of BMI and other body composition metrics [26,27].

BMI-for-age and sex reference values and charts have evolved since the initial proposal in 1982. As of the year 2025, international growth reference charts displaying BMI-for-age and sex smoothed *z*-scores or percentile cutoffs are still used to categorize children and adolescents into underweight, healthy or normal weight, overweight, obesity, and severity of obesity (terminology and cutoffs may vary depending on the chart) [28]. The 4 most commonly used international growth references for classifying children and adolescents with overweight and obesity, based on BMI-for-age and sex, are as follows: *1*) the 2000 US Centers for Disease Control and Prevention (CDC) growth charts reference for the United States of America for individuals aged between 2 and 20 y [3,29] as well as the more recent CDC’s Extended BMI-for-age growth charts to monitor BMI values beyond the 97th percentile [4], *2*) the 2000 International Obesity Task Force (IOTF) reference for individuals aged between 2 and 18 y [5,6,30], *3*) the WHO 2006 child growth standards for children aged under 5 y [7,31], and *4*) the WHO 2007 growth reference for children and adolescents aged between 5 and 19 y [8,32]. These international references are often used along with country-specific national references to more accurately reflect the characteristics and body composition of that country’s population [[33], [34], [35], [36]]. Due to the complexity and variability in defining and measuring obesity, it was decided by the research team in consultation with the WHO that an expert consultation was needed to ensure that our DTA of BMI-for-age and sex was reflective of the highest quality research available.

## Expert Consultations and Summary Development

We identified experts by searching authors of articles published from 2010 to January 2024 in Scopus with the following keywords: obesity, pediatrics, and nutrition. Similar searches were conducted in PubMed, Google Scholar, the WHO’s Global Information database with an extended group of keywords related to obesity diagnosis, body composition and body fat measures, anthropometry, and metabolic syndrome focusing on children and adolescent populations. The keywords were translated into local languages of the journal if possible. We assessed each author on relevant publications, with priority on the first, last, and corresponding authors, within the selected publications collecting the following generic parameters: main areas of expertise, country and institution, common topics of interest from other published articles, and other relevant experience described on ResearchGate, Scopus author database, Open Researcher and Contributor ID, Google Scholar, and various institutional websites.

With this process, 128 experts from 50 countries were identified (RXM) and contacted to participate in the expert consultation meetings by our research team (LEO). Experts were asked to provide written feedback on 4 questions (Table 1) related to protocol development for our upcoming DTA SR and were invited to join 1 of 2 moderated virtual group discussions held in January 2024 (moderated by LEO and LL-P). Individual meetings were held for those who indicated interest in participation (by LEO, RXM, and LL-P) but who could not join either of the 2 group discussions. Experts were contacted ≤3 times but were not asked the reason for declining or not responding to the inquiry.

**TABLE 1:** Four questions posed to experts to inform key decisions for the DTA SR protocol.

Decision for protocol	Question posed to experts
1. Reference standards	What are the best available methods or combinations of methods to use as reference standards for measuring body composition for infants, children, and adolescents?
2. Index tests	What clinical tests or combinations of clinical tests do you consider to assess body composition and adiposity for infants, children, and adolescents, other than BMI?
3. Thresholds for determining excessive adiposity	What are the recommended clinical adiposity cutoffs for determining obesity for infants, children, and adolescents?
4. Types of adiposity	What are the types of adiposity (total or regional adiposity, adipose cell type, size, or count) critical for determining obesity for infants, children, and adolescents?

Abbreviations: DTA, diagnostic test accuracy; SR, systematic review.

For those that agreed to participate, they were first asked to provide written feedback on the 4 questions listed in Table 1 prior to the group discussion meetings. Then, during the group discussions, experts were asked to participate in 8 polls via Zoom, if able (poll questions are described in Supplemental materials). An open discussion period was held after each poll in which experts were encouraged to share their perspectives or rationales for their responses if provided. Experts were also encouraged to speak freely throughout the entirety of the meeting to provide their expertise as they felt appropriate.

Experts’ written and oral responses are summarized narratively in this report. The summary was supplemented with additional literature provided by experts or identified by our team members as needed to provide a balanced presentation of the topic. Systematic literature searches were not performed for this summary. A draft of this summary report was circulated to all invited experts, including both those who did or did not participate, to ensure accuracy and solicit additional feedback. In addition, the draft was posted for open public comment on the Texas A&M Agriculture, Food, and Nutrition Evidence Center website for 1 mo in July 2024 (https://evidencecenter.tamu.edu/assessing-excess-adiposity-in-children-and-adolescents/). The public comment period was advertised in a scientific poster at the American Society for Nutrition’s Nutrition 2024 conference in Chicago, IL [37] as well as through the Evidence Center’s LinkedIn page. Several experts provided additional references during the feedback period to support statements made during the group meetings. No public comments were received.

## Expert Feedback

**FIGURE 1:**
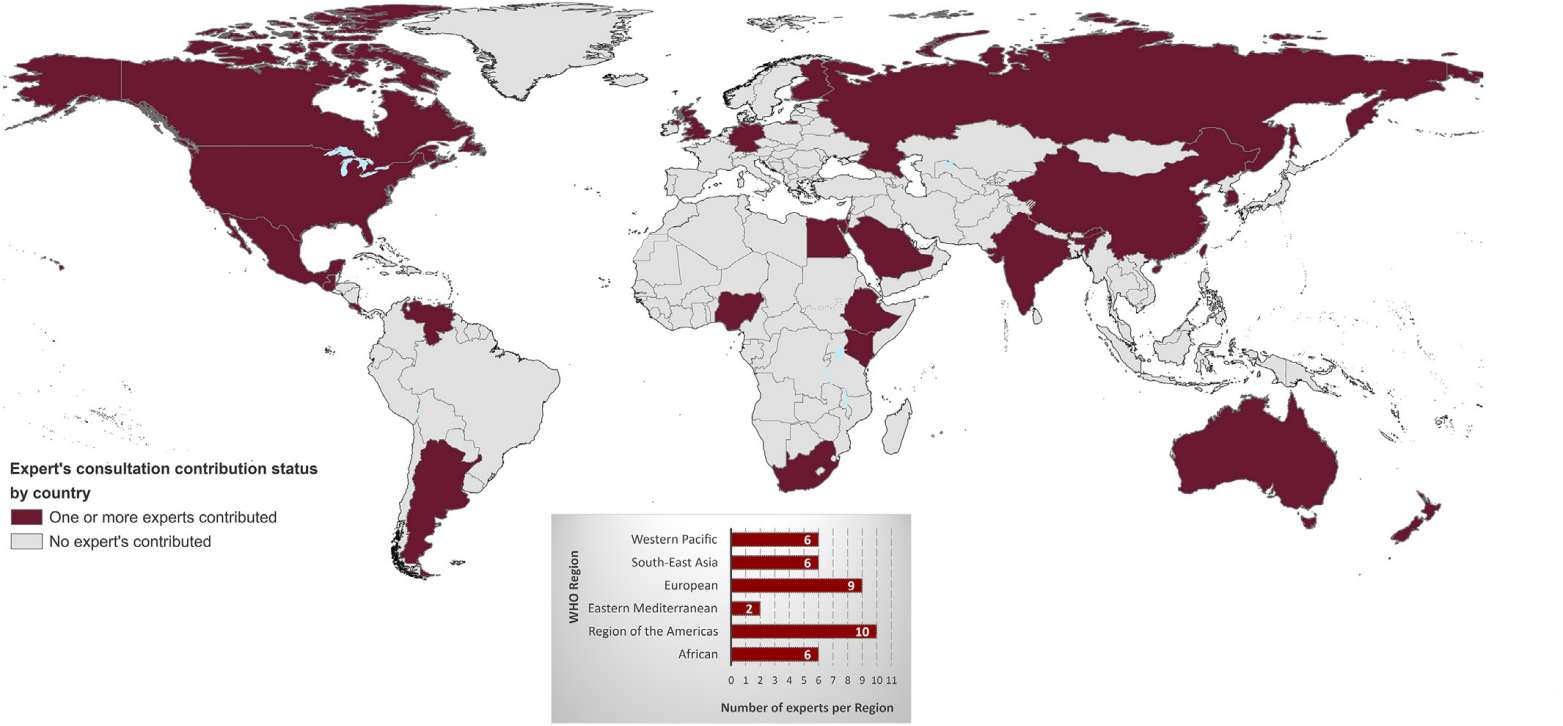
Map of represented countries with ≥1 expert that provided written feedback and/or attended an expert consultation meeting; *n* = 23 countries represented by 39 experts. The boundaries and names shown and the designations used on this map do not imply the expression of any opinion whatsoever on the part of the WHO concerning the legal status of any country, territory, city or area or of its authorities, or concerning the delimitation of its frontiers or boundaries. Dotted and dashed lines on maps represent approximate border lines for which there may not yet be full agreement.

A total of 39 experts provided written or oral feedback, 25 attended the group meetings, and 23 of those 25 experts participated in the live polls in the meetings. Of these 23, 52% were generally familiar with DTA frameworks whereas 48% had heard of but were not familiar with the details of the methodology (Supplemental Figure 1). Additionally, one-on-one meetings were held with 6 experts who could not attend the group discussions. All expert contributors, from 23 countries representing all 6 WHO regions (Figure 1), are listed in the Acknowledgments.

## Decision 1: Reference Standards

A reference standard is described by Cochrane as the best available method of determining whether a person has the target condition of interest or not [38]. For our DTA SR, we defined “best” as the most accurate and reliable methods, independent of cost or feasibility, that elicit the least amount of bias in comparison with the index test(s) under investigation. Experts were asked, “What are the best available methods or combinations of methods to use as reference standards for measuring body composition for infants, children, and adolescents?” The goal of this question was to solicit feedback on acceptable methods (including required machines or equipment) to use as reference standards for measuring body composition for infants, children, and adolescents to inform the inclusion and exclusion criteria for our DTA SR. The experts recommended dual energy X-ray absorptiometry (DXA), hydrostatic underwater weighing, air displacement plethysmography densitometry (ADP), MRI, deuterium oxide dilution (D_2_O), and multicompartmental models (MCM) using 1 or a combination of these methods as acceptable reference standards for assessing body composition at 0–19 y of age. Strengths and limitations of these methods are available in Table 2 [[39], [40], [41], [42], [43]] and described previously [40, 43].

**TABLE 2:** Strengths and limitations of reference standards used for diagnostic test accuracy studies to measure body composition and obesity in infants, children, and adolescents.

Method	Strengths	Limitations
Hydrostatic weighing (densitometry)	• Two-compartmental model (fat mass + fat-free mass)	• Uncomfortable, impractical, and time consuming • Nonportable, expensive equipment, training, and equipment maintenance
Air displacement plethysmography (densitometry)	• Reference standard for infants at different stages of development are available for high-income countries • Two-compartmental model (fat mass + fat-free mass) • Relatively easy measure for infants because the machine (e.g., PEA POD) not affected by movement, defecation, or urination • Smallest bias and highest limit of agreement [39]	• Needs repeated calibrations and may be uncomfortable [40] • Procedural limitations due to the behavior and continuous movement of children of this age [40] • Nonportable, expensive equipment, maintenance, and training; sensitive to software updates and technological advancements • Cannot determine location or distribution of fat • Cultural insensitivities in that removal of clothing and facial or head coverings are required • Lack of robust and in-depth validation studies, and few studies reporting on sources of error even unknown, and random [40] • Not validated for under 2 y of age [40]
Deuterium oxide dilution (total body water)	• Smallest bias and highest limit of agreement [39] • Portable; samples can be frozen and shipped for analysis • Assumes a standardized hydration status that may not be appropriate for all age groups and developmental stages	• Time consuming • Expensive isotope, equipment maintenance, and training
Dual energy X-ray absorptiometry (DXA or DEXA)	• Most sensitive compared with other reference standards in diagnosing obesity [41] • Can provide assessment of regional adiposity (arms, legs and trunk), but normative values unavailable [40] • Three-compartmental model (fat mass + fat-free mass + bone mass) • Smallest bias and highest limit of agreement [39]	• Nontransportable and expensive equipment, extensive training, and equipment maintenance; can be uncomfortable/difficult to stay still and measures are affected by movement [40] • Centiles for OW and OB diagnosis vary and are not standardized (often, but not always age- and sex-adjusted) • Sensitive to software updates and technological advancements • High variability due to different calibrations across all DXA systems both within and across manufacturers [40] • Radiation exposure [40] • Lack of robust and in-depth validation studies, and few studies reporting on sources of error even unknown, and random [40] • Can overestimate fat mass compared with 4 compartmental model [42]
MRI	• Considered a gold standard of body composition measures for accuracy [43] • Measures whole body and regional distribution of fat mass, lean mass, and bone skeletal muscle quantification • Accurate quantification of body composition at the tissue and organ level • Three-compartmental model (fat mass + fat-free mass + bone mass)	• Nontransportable and expensive equipment, maintenance, and extensive training • Can be uncomfortable or claustrophobic • Measures are affected by movement and crying and may require sedation in infants and children [40] • Time consuming • High-cost and significant technical examiner expertise [40]
Four compartmental model	• Fat mass + fat-free mass + bone mass + total body water) • Not affected by hydration status because it is directly measured, rather than predicted or estimated • Considered the gold standard for infants, children, and adolescents and often used as a reference standard [39,41,43] • Can be calculated using multiple or single methods, including DXA, MRI, and/or isotopes	• Time consuming • Expensive • Several different combinations of measurements (DXA, BIA, ADP, labeled water dilution) and varying predictive equations

Abbreviations: ADP, air displacement plethysmography densitometry; BIA, bioelectrical impedance; OB; OW.

Although all these methods are acceptable reference standards for our DTA SR, there was consensus that MCM, D_2_O and MRI are the most reliable and accurate methods to measure adiposity, independent of cost and feasibility, for children and adolescents (Supplemental Figure 2). Most experts agreed that MCMs, particularly 4-compartmental models that divide total mass into fat mass, fat-free mass, bone mass, and total body water, calculated using a combination of these methods, were preferable [42]. Although D_2_O methods are useful in free-living populations or for in-field setups with limited burden on the participants [44], it is the only method that does not assess regional adiposity. For children aged younger than 2 y, ADP was the preferred method because it can accommodate infants as small as 8 kg and movement does not affect the measurement as it would for DXA, hydrostatic underwater weighing, or MRI [43]. On the basis of this information, the experts suggested stratifying the meta-analysis in the DTA SR by reference standard to assess whether the accuracy of BMI for diagnosing obesity may be influenced by the quality of the reference standard (Table 2) [[39], [40], [41], [42], [43]].

The experts noted that although the methods described above are the strongest choices as reference standards, they are not without limitations. First, they are proxy measures of body composition given that the only direct measure of body composition is autopsy dissection. Second, each of the methods uses predictive “black box” algorithms to estimate body composition that can introduce bias and variability [45]. It is important to understand what anthropometric input variables are used in predictive algorithms because of their likely correlation with the index test of interest (BMI-for-age and sex) in DTA studies. Third, different types and sources of variability or error can be introduced to an analysis depending on the machine or software version used which would be difficult to account for in a DTA SR. Finally, the methods are expensive and may be unobtainable in a clinical setting or lower resource areas. Thus, there may be a paucity of DTA studies that use these methods as reference standards in lower resource countries which may result in biased literature search results. However, these are the best available methods for reference standards and these limitations will be made clear in the conclusions and discussion of the upcoming SR.

DXA is often referred to as a "gold standard" for assessing body composition. However, there was discussion about whether DXA is superior to all other methods or if it is simply more conventional due to relatively lower cost, resources, and training required [40]. DXA poses additional measurement challenges for children aged under 5 y. First, radiation exposure limits the number of safe repeated measures and second, young children may find it difficult to be still, which can induce error in the measurement [40]. A notable advantage of DXA is the ability to calculate MCM, because DXA estimates bone mineral content, regional adiposity (arms, legs, trunk), fat mass, and bone-free lean mass [40]. However, the use of these body compartments may vary by machine, software, or algorithm.

In some DTA SRs, a clinical diagnosis is used as a reference standard in which a healthcare provider decides which tests to administer and judges the results based on their expertise to diagnose the target condition. The experts that we consulted questioned the suitability of including articles that use a clinical diagnosis performed by a healthcare provider as a reference standard for a DTA SR of BMI-for-age and sex. Most advised against it because BMI-for-age and sex is a common screening and diagnostic test used by healthcare providers to diagnose obesity which would introduce bias. Further, children with a higher BMI are more likely to be under evaluation for high risk of obesity, increasing risk for confounding by indication [46]. There would also be considerable variability in clinical diagnoses as there is not 1 standardized algorithm used globally, or even regionally, by healthcare providers to diagnose obesity. Interestingly, however, several experts said anecdotally that a visual inspection can often suffice to decide if the child has a concerning amount of excess adiposity.

## Decision 2: Index Tests

An index test is the test under investigation in the DTA framework. In our DTA SR, the primary objective is to assess the accuracy of BMI-for-age and sex (main index test) for the diagnosis of obesity for children and adolescents. As secondary objectives, we will also be assessing the accuracy of other index tests or combinations of index tests in diagnosing obesity for infants, children, and adolescents. A preliminary list of potential index tests was provided to experts. They were asked, “What clinical tests or combinations of clinical tests do you consider to assess body composition and adiposity for infants, children, and adolescents, other than BMI?” and asked to provide any additional index tests. The goal of this question was to solicit feedback to ensure a comprehensive literature search. All index tests suggested by experts to use with or without BMI-for-age and sex are listed in Table 3 [23,[47], [48], [49]].

**TABLE 3:** Reference standards and index tests used for assessing excessive adiposity that can impair health for infants, children, and adolescents^1^.

Reference standards	
• Dual-energy-X-ray absorptiometry • Hydrostatic underwater weighing • Air displacement plethysmography densitometry	• MRI deuterium oxide dilution • Multicompartmental models using 1 or a combination of these methods
Index tests used alone^2^	
• BMI (weight/height^2^)-for-age and sex • Waist circumference • Waist-to-height ratio • Waist-to-hip ratio • Weight-for-height/weight-for-length • Skinfold thickness measures (triceps, biceps, subscapular, suprailiac for sex and age) • Mid-upper arm circumference • Neck circumference • Triponderal mass index/ponderal index/Rohrer index (weight/height^3^) • Relative weight • Digital anthropometry • Body volume index • Ultrasound • Bioelectrical impedance analysis	• Percentage of expected or ideal body weight using Harvard growth standard • 3D optical scans and mobile applications • Metabolic load-capacity model or index (the ratio of fat mass or adipose tissue to fat-free mass) • Benn Index (weight/height^p^)^3^ • Peripheral subcutaneous fat/peripheral adiposity index • Central subcutaneous fat/central adiposity index • Relative fat mass • Emerging fat mass prediction models proposed in the literature based on various types and combinations of demographics, anthropometrics, and clinical health data (see references [[47], [48], [49]] for examples)
Index tests used in combination with BMI-for-age and sex^4^	
• Waist circumference • Waist-to-height ratio • Waist-to-hip ratio • Skinfold thickness measures • Neck circumference • Body fat percentage or fat mass index computed with one of the index test methods listed above. • History of maternal gestational diabetes • History of fetal macrosomia	• Metabolic measures: fasting blood glucose, total cholesterol, triglycerides, LDL, HDL, insulin, leptin, inflammatory markers, blood pressure, etc. • Weight gain trajectory • Adverse in utero environment • Gestational diabetes history • Intrauterine growth restriction and small for gestational age • Genetic testing • Family history of metabolic complications or bariatric surgery

Abbreviations: 3D, three dimensional; BIA, bioelectrical impedance; FMI, fat mass index; WC, waist circumference; WHtR, waist-to-height ratio.

1 This curated list of reference standards and index tests is based on written and oral feedback from experts, including during group and individual discussions, as well as live polls administered during the group discussions. Tests are listed in no particular order.

2 Of the total 39 experts, 20 participated in a poll to select the most feasible, reliable, and accurate index tests for assessing adiposity (Supplemental Figure 3). The following tests, aside from BMI-for-age and sex, had ≥2 votes: WC, BIA, FMI, skinfold thickness, WHtR, weight-for-length/weight-for-length, triponderal mass index, ultrasound, and WHR. Waist circumference (60% of experts chose) and BIA (45% of experts chose) were the top 2 choices.

3 The appropriate power of height "p" is determined by regressing log weight-for-age on log height-for-age, giving an index of relative weight which is highly correlated with weight but uncorrelated with height for age [23].

4 Of the 39 experts, 22 participated in a poll to select the top 3 most feasible, reliable, and accurate tests to assess adiposity in combination with BMI-for-age and sex to improve the accuracy of obesity diagnosis for children (Supplemental Figure 4). The following tests received ≥2 votes: waist circumference, BIA, body fat percentage, FMI, WHtR, skinfold thickness measures, metabolic risk factors, weight gain trajectory, and gestational diabetes history of the mother.

The experts identified 2 preferred index tests, waist circumference (WC) and bioelectrical impedance (BIA), for use alone or in combination with BMI (Supplemental Figure 3 and 4). WC was preferred, but only some countries use it for clinical diagnosis of obesity. For example, in India, WC is informative because the population tends to have lower muscle mass and higher abdominal adiposity and metabolic risk factors at lower BMIs compared with European or North American populations [50,51]. WC for these latter populations is almost perfectly correlated with BMI-for-age and sex; thus, the experts suggested that there is little added value of these tests beyond BMI [52,53]. BIA was the second most preferred by experts because it is a simple tool available in clinical or low-resource settings. Segmental BIA [54], which can predict regional adiposity, is used in combination with BMI to increase the accuracy of obesity diagnosis. Newer multicurrent BIA devices have fewer limitations than single-current devices, such as errors from hydration status. Therefore, advancements in technology as well as simplicity, cost, and convenience outweigh historical concerns in the variability of prediction equations and accuracy of BIA for children and adolescents [11,[55], [56], [57]]. Experts said that sometimes in their own practice, WC, BIA, and BMI-for-age and sex are used in combination for obesity diagnosis in children and adolescents, with or without the addition of other index tests listed in Table 3 [23,[47], [48], [49]].

We also expect to identify literature about novel predictive models that are used as index tests for diagnosing obesity (Table 3) [23,[47], [48], [49]]. During the expert consultation, the Hudda model was mentioned on multiple occasions. This model was introduced in 2019 and combines height, weight, age, sex, and ethnicity to predict body fat mass and discriminate between lean and fat mass [[47], [48], [49]]. This model was developed with the intention to improve the accuracy of BMI-for-age and sex to predict excess adiposity using data from 19 countries with children aged 4–15 y [47,49]. As with many index tests and predictive models, there are concerns whether these add value in the diagnosis of obesity beyond BMI-for-age and sex alone [48,58] and whether sex, age, and ancestral background are appropriately considered in calculations [[59], [60], [61]].

It was recognized throughout the discussion that almost any of these tests will identify individuals who are at the highest risk of obesity and are in most need of intervention. Experts mentioned that from their perspective and experience, misdiagnosis tends to happen more toward the middle of the distribution curve or for those who may not fit the typical phenotype of obesity. Notably, there were few suggestions for index tests to use for children aged 0–2 y. Rather than BMI, weight-for-height or weight-for-length is conventionally used to assess body composition in children aged 0–2 y. However, these measures do not account for variations in body structure and composition [23, 58]. Like the reference standards described previously, many of these tests can be biased by the assessor, machine, software, and predictive algorithm used [62]. This variability will be explored in subgroup analyses in our upcoming DTA SR.

## Decision 3: Thresholds for Determining Excessive Adiposity

As demonstrated by a previous SR [39], adiposity cutoffs used for determining obesity vary throughout the literature depending on the population or index test of interest. Approaches used to estimate cutoffs include but are not limited to percentiles, *z*-scores, and absolute thresholds, with different cutoffs within each approach [39]. The cutoffs are commonly influenced by age, sex, race/ethnicity, and puberty status. To better understand this inconsistency, experts were asked, “What are the recommended clinical adiposity cutoffs for determining obesity for infants, children, and adolescents?” The goal of this question was to solicit feedback concerning acceptable and unacceptable thresholds used for the reference standard measurements at either the international, national, or local level.

When asked about acceptable cutoff points, ranges, and/or percentiles to assess adiposity using total body fat, about half of the experts indicated a preference for total body fat percentiles by age and sex, specific to the country or ethnicity under study (Supplemental Figure 5). About one-third of the experts voted that they do not recommend the use of total body fat as a measure for the reference standard. About half of the experts voted that >25% of total body fat for boys and >30% of total body fat for girls were acceptable cutoff ranges to assess adiposity (Supplemental Figure 5). However, during discussion, others discouraged the use of these fixed thresholds which have been widely used in the past [63]. This discussion led to a larger consensus (∼75% of experts who voted) to not fix thresholds (Supplemental Figure 6) but rather percentiles that are dependent on age and sex. Most experts agreed that pubertal status, marked by significant changes in body fat composition and distribution, should also be considered when using these cutoffs but there was little input about the appropriate approach.

Some (38%) experts preferred the use of the fat mass index (FMI) [59,64,65] instead of body fat percentage [66] (Supplemental Figure 7) as the preferred measure to use from the reference standard methods. Reasons for this included *1*) percent body fat is not a sensitive measure to changes in body composition because fat mass is included in both the numerator and denominator, *2*) percent fat mass is not a good indicator of nutritional status, *3*) percent fat mass could be reflective of either high fat mass or low lean mass [53]. Further, FMI is easily compared with fat-free mass index (equal to fat-free mass/height^2^ sometimes referred to as lean mass index) and BMI because it uses the same units (kg/m^2^) and is closely related to other factors such as age [15]. Age is important to consider with these measures because excess adiposity is less common at younger ages, and young children within the higher BMI percentiles tend to have lower FMI than older children within the corresponding BMI percentile for their age group. For example, adolescent boys at a BMI of ∼20 have ∼18% body fat whereas younger boys at that BMI level have ∼30% body fat [15]. This is also demonstrated by BMI charts where mean reference values for boys are 14 at 5 y old and 16 at 14 y according to the CDC BMI-for-age percentiles sex [3]. There was discussion about what an appropriate cutoff for FMI may look like for this age group. More stringent cutoffs may be required for children younger than6 y for FMI matching the WHO BMI recommendations for 0–5 y [15] to avoid overestimation of obesity prevalence in this age group.

It was also suggested that adiposity trajectory, changes over long periods of follow-up, and adiposity rebound should be considered when using these tools to determine appropriate obesity thresholds [67]. Adiposity trajectory can also inform the associated risk of obesity and related metabolic disease in adulthood. Importantly, assessing adiposity trajectory or age of adiposity rebound requires measures that are readily accessible, such as height and weight. Modern population samples can be used to assess these longitudinal metrics. However, experts emphasized that reference population samples used to determine percentiles and thresholds should be from before the obesity epidemic to establish a baseline for normal growth patterns in the absence of the influence of increasing obesity rates. Many countries do not have datasets that predate this era which may be a bias that we encounter in the literature.

Most of the countries within the 6 WHO regions [68] rely on the WHO, US CDC, or IOTF references and standards to determine cutoffs for obesity diagnosis, though these charts are not relevant for all world areas, races, ethnicities, or age groups [65,69]. Some countries have their own cutoffs that are more specific to their populations for BMI [[70], [71], [72]], WC [[73], [74], [75], [76]], waist-to-height ratio [[77], [78], [79]] or other measures of adiposity such as BIA [80,81] or DXA [82]. For example, the WHO BMI-for-age and sex cutoffs underestimates excessive adiposity compared with the deuterium dilution method for children from African countries [12,83]. However, country-level cutoffs may still not be specific enough; local cutoffs may be more appropriate. For example, tribal communities can have different body composition and fat distribution compared with nontribal communities within the same country [84]. It is also important to consider immigration status. For example, those who immigrate to Israel tend to present with adverse metabolic risk factors at a lower BMI compared with those who were born in that country [85,86]. Similarly, children who identified as Black who immigrated to the United States compared with children who identified as Black born in the United States had different BMI trajectories [87]. We will consider both international and national thresholds as the data allow us to represent these noted variations across demographic and geographic populations in our DTA SR.

Another limitation of the international growth charts is that the prevalence of obesity is assumed to be the same across the age categories, which is not accurate as obesity risk increases with age [15,[88], [89], [90]]. For example, BMI is a better indicator of lean mass rather than fat mass for younger age groups [15]. This suggests that the BMI cutoff for younger age groups should be appreciably higher than that of the older age groups, similar to the WHO BMI-for-age and sex approach for those under 5 y old (e.g., the WHO overweight category is defined by >1 *z*-score for children older than 5 y and >2 *z*-scores for children <5 y sex [7]). Obesity in preschool-aged children would be overestimated without a higher cutoff, leading to them receiving unnecessary treatment and misdirecting resources and efforts away from those at higher risk, including older children and adolescents. Additionally, some children who are classified as overweight at <5 y old may not remain overweight through adolescence or adulthood [26,91]. As children age, consideration of pubertal stage and sex becomes particularly important. For example, data from the WHO MultiCenter Growth Reference Study showed that there were differences between sexes in FMI by as early as 24 mo of age [92] and body fat in boys continues to decrease after puberty onset whereas it increases in girls [81]. It is conventional to categorize by age, then sex (e.g., BMI-for-age and sex), but it was suggested that the reverse order may be more appropriate, and is what occurs in practice, in deciding where the child falls within the standardized distribution. Overall, there was general agreement that age, sex, race, ethnicity, and pubertal stage should be considered in using these methods to determine thresholds or cutoffs for diagnosing obesity. However, there was no resolution or consensus on the approach.

## Decision 4: Types of Adiposity to Consider

It is important to understand which reference standards can assess total compared with regional adiposity and any potential bias when comparing these measures to BMI-for-age and sex or other index tests. Therefore, experts were asked, *“*What are the types of adiposity (total or regional adiposity, adipose cell type, size, or count) critical for determining obesity for infants, children, and adolescents?” The goal of this question was to determine the importance of adiposity localization or distribution (i.e., regional adiposity) for obesity diagnosis in children and adolescents worldwide, both for clinical practice and research.

Most experts (88%) agreed that both total and regional adiposity (i.e., accumulation of adipose tissue in certain areas such as central, visceral, abdominal or subcutaneous) are important for diagnosing, monitoring, and/or treating obesity (Supplemental Figure 8) because of cardiometabolic health risks associated with central obesity. For example, a recent study using DXA-measured central adiposity for those aged 15–24 y predicted a 21% risk of insulin resistance whereas DXA-measured total body fat predicted an 11% risk of insulin resistance [93]. However, accurately measuring regional adiposity, with an emphasis on central adiposity, remains a challenge in infants, children, and adolescents. Experts expressed frustration at the limitations of the current methods for assessing total adiposity for this population (Table 2) [[39], [40], [41], [42], [43]]. Therefore, they felt that difficulties in accounting for age, sex, race, ethnicity, or puberty status in measuring total adiposity are only amplified when measuring central adiposity. It was also noted by several experts that the importance of lean mass is often overshadowed by the focus on fat mass. Higher muscle mass, comprising highly metabolically active tissue, is often associated with better metabolic outcomes and reduced risk of disease.

There was little discussion on adipose cell type, size, or count, other than that most experts agreed that these measures are not relevant for a clinical diagnosis of obesity. Adipose cell count and size can be assessed in the research setting but requires an adipose tissue biopsy which is invasive and not appropriate for children and adolescents unless collected during a necessary medical procedure.

## Limitations

We convened experts across the globe to share their expertise on assessing body composition and obesity in infants, children, and adolescents to inform the development of our DTA SR protocol about the accuracy of using BMI to diagnose obesity in this age group. However, there were some limitations in our approach. First, although we successfully convened experts across 23 countries representing all 6 WHO regions, the response rate was low from those initially contacted. Second, few experts had experience using these measures or diagnosing obesity in infants. Therefore, a decision was made after the consultation to exclude infants in the upcoming DTA SR of BMI-for-age and sex. It will be required to convene a future expert panel to inform a protocol specific to infants which would likely focus on weight-for-length or weight-for-height as the index tests of interest. Third, we did not conduct systematic literature reviews to support this summary as our objective was to focus on expert opinion.

## Expert-Informed Decisions for the DTA SR Protocol Development

**FIGURE 2:**
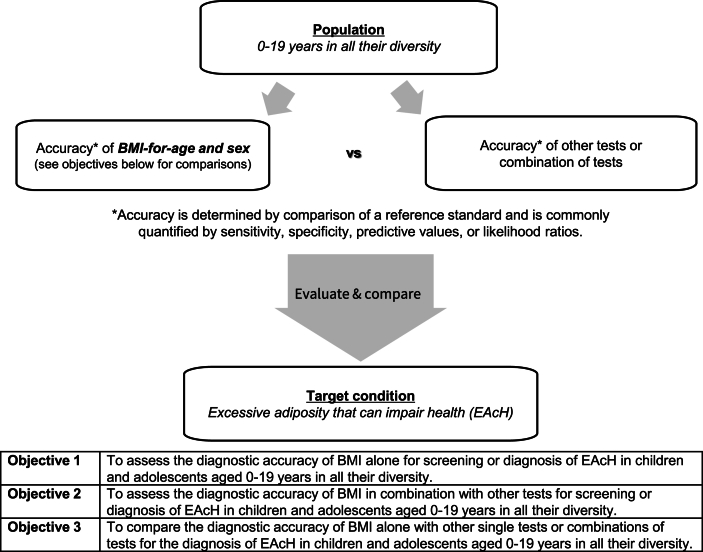
Framework and objectives for a diagnostic test accuracy systematic review of BMI-for-age and sex for assessing excessive adiposity that can impair health for children and adolescents in all their diversity.

The framework and objectives for our upcoming comparative DTA SR of BMI-for-age and sex are shown in Figure 2. The expert consultations significantly informed several key decisions that are reflected in Figure 2 and in the published protocol. First, DXA, hydrostatic underwater weighing, ADP, MRI, D_2_O, or MCMs will be considered as acceptable reference standards. Use of these reference standards to measure either fat mass/height^2^ or percent body fat, but not fat mass alone, for either total or central adiposity will be included in our analysis. All thresholds used to diagnose excessive adiposity with these measures from reference standards must consider both sex and age or maturation stage. Second, for secondary aims, we will assess available data on the accuracy of other index tests or combinations of index tests listed in Table 3 [23,[47], [48], [49]]. All reference standards and index tests listed in Table 3 [23,[47], [48], [49]] were included in our comprehensive database search strategy to ensure high sensitivity of article retrieval. Finally, we will include studies that assess total and central adiposity, but not adipose cell type, size, or count to ensure that the generalizability of our findings is relevant for a clinical setting.

For our expert convening in January 2024 and throughout the protocol development process, we relied on the WHO’s definition of obesity at that time which is a “chronic complex disease defined by excessive adiposity that can impair health” [28]. A year after our expert meetings were held, Lancet released new obesity definitions and diagnostic frameworks in January 2025. The Lancet framework uses other measurements of body size in addition to BMI to define either clinical (defined as a systematic disease caused by excess adiposity) or preclinical obesity (defined as excess adiposity without current organ dysfunction or physical limitations, but with future health risk) [94]. One motivation behind the new Lancet definitions was that the term obesity was often conflated with BMI because obesity was conventionally defined by this 1 single metric [95]. We did not adopt Lancet’s new definition of obesity for our protocol because our protocol development process began prior to the release and the acceptability of Lancet’s definition by researchers and clinicians around the world was unclear at that time. Therefore, our research team designed the protocol to assess the DTA of “excessive adiposity that can impair health” (EAcH), rather than using the term obesity. To define “health” in EAcH, we use the WHO’s International Classification of Functioning, Disability and Health [96,97].

In conclusion, engagement from global experts in the field of childhood obesity and body composition informed critical decisions for an SR of the accuracy of BMI-for-age and sex for diagnosing excessive adiposity that can impair health in children and adolescents. The protocol for that review is now published (https://osf.io/vzwjt). The findings of this upcoming DTA SR will inform the use of BMI and/or other diagnostic measures for the assessment of excessive adiposity for the WHO’s living guidelines for the integrated management of children and adolescents with obesity in all their diversity.

## Author contributions

The authors’ responsibilities were as follows – LEO, LL-P, RXM: convened the meeting, summarized the responses, and wrote the article; MS, AJM: had primary responsibility for final content; and all authors: designed research, and read and approved the final manuscript.

## Data availability

Data described in the manuscript, which includes written feedback and transcripts of group discussions, can be made available on request as appropriate.

## Funding

Texas A&M Agriculture, Food, and Nutrition Evidence Center. Juan Pablo Peña-Rosas is a full-time staff member of the WHO. The authors alone are responsible for the views herein expressed and they do not necessarily represent the views, decisions, or policies of the institutions with which they are affiliated.

## Conflict of interest

The authors report no conflicts of interest.
