# Supplementary material for: Report of a Meeting: An Expert Consultation on Body Composition and Adiposity for Children and Adolescents in All Their Diversity
Source: Curr Dev Nutr. 2025 Jun 3;9(7):107475. doi: 10.1016/j.cdnut.2025.107475 (PMC12269979; doi:10.1016/j.cdnut.2025.107475)
Supplement: multimedia component 1 [file mmc1.docx]

Contents

[Supplementary Figure 1 2](#_Toc176947390)

[Supplementary Figure 2 3](#_Toc176947391)

[Supplementary Figure 3 4](#_Toc176947392)

[Supplementary Figure 4 5](#_Toc176947393)

[Supplementary Figure 5 6](#_Toc176947394)

[Supplementary Figure 6 7](#_Toc176947395)

[Supplementary Figure 7 8](#_Toc176947396)

[Supplementary Figure 8 9](#_Toc176947397)

# Supplementary Figure 1

Poll 1: Are you familiar with reviews of diagnostic test accuracy studies?


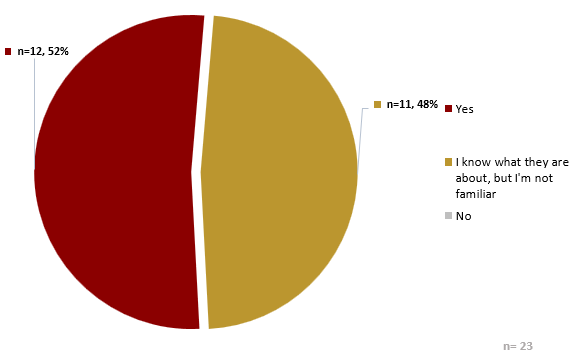


A total of 39 experts provided written or oral feedback, 25 attended the group meetings, and 23 of those 25 experts participated in the live polls in the meetings.

# Supplementary Figure 2

Poll 2: Please rank the direct methods to measure adiposity from best (1) to worst (5) method (1), without consideration of practicality or cost (options are listed in Table 2).


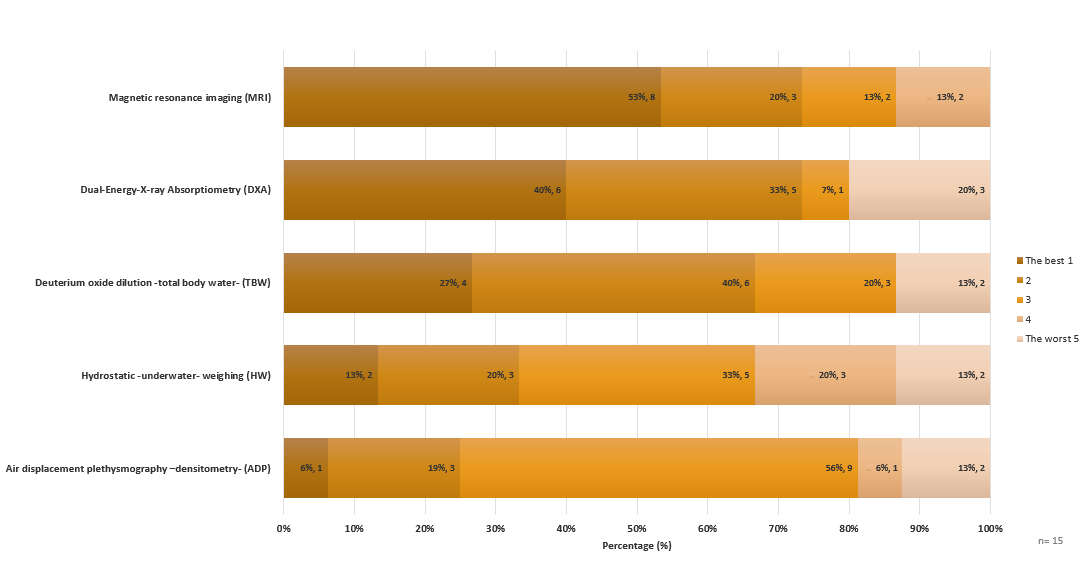


A total of 39 experts provided written or oral feedback, 25 attended the group meetings, and 23 of those 25 experts participated in the live polls in the meetings.

# Supplementary Figure 3

Poll 3: Select from the list below (listed in Table 2) the 3 most feasible, reliable and accurate measures to assess adiposity for diagnosing obesity in children, besides BMI.


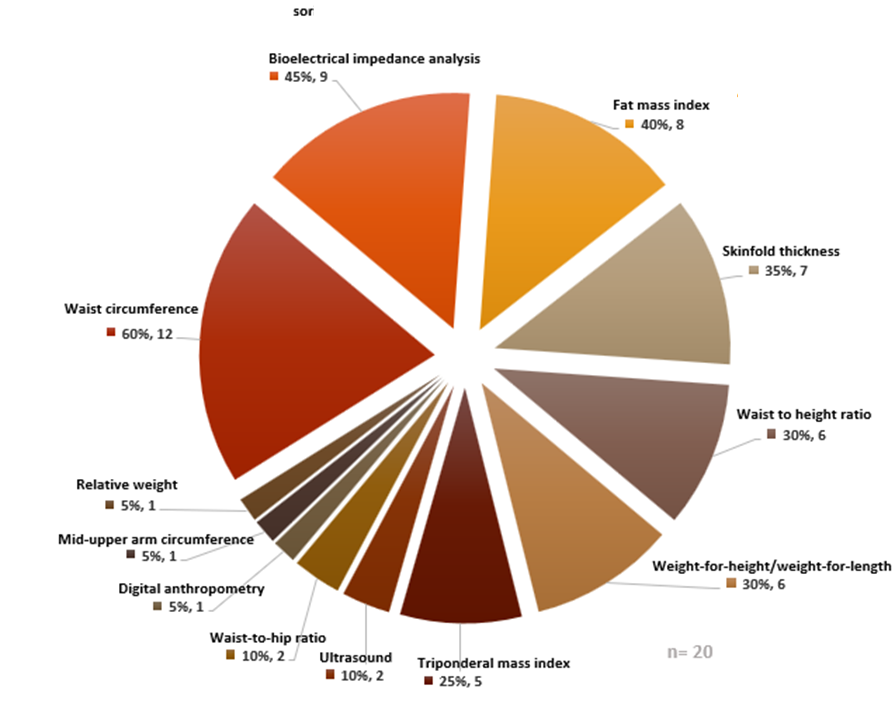


A total of 39 experts provided written or oral feedback, 25 attended the group meetings, and 23 of those 25 experts participated in the live polls in the meetings. The percent refers to the percent of responders that chose that method out of 20 responders. The number following the percent is the number of responders that chose that method.

Some measurements, such as Peripheral subcutaneous, Neck circumference, Metabolic load-capacity model, Fat (peripheral adiposity) index, Expected or ideal body weight (Harvard growth standard), Central subcutaneous fat (central adiposity) index, Body volume index (BVI), Benn index and 3D optical scans and mobile applications, were not included in the graph due to receiving a score of 0.

# Supplementary Figure 4

Poll 4: Comparator(s) test (2). Select from the list below the 3 measures that you think should be used in combination with BMI-for-age and sex for the most feasible, reliable and accurate assessment of adiposity for diagnosing obesity in children?


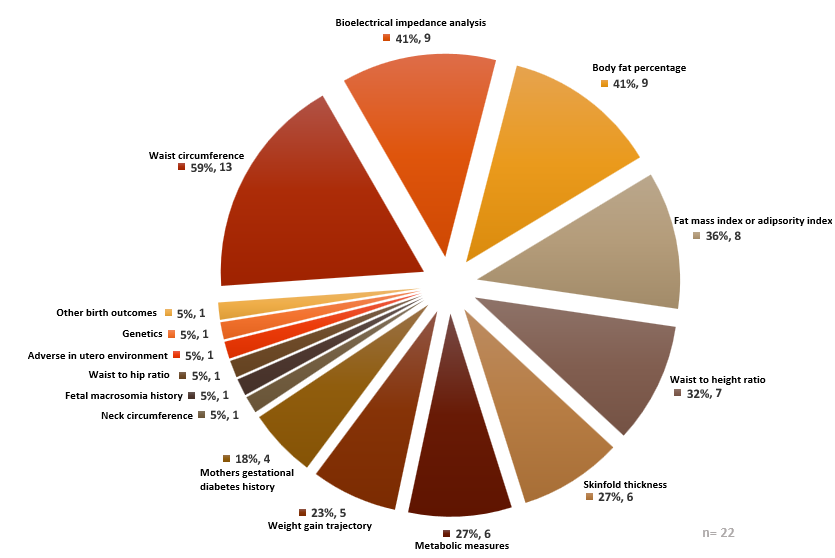


A total of 39 experts provided written or oral feedback, 25 attended the group meetings, and 23 of those 25 experts participated in the live polls in the meetings. The percent refers to the percent of responders that chose that method out of 20 responders. The number following the percent is the number of responders that chose that method. *The measure Family history of metabolic complications/bariatric surgery was not included in the graph due to receiving a score of 0 . Birth outcomes included small for gestation age and intrauterine growth restriction.*

# Supplementary Figure 5

Poll 5: Which are acceptable cut-offs ranges and/or percentiles to assess adiposity for obesity diagnosis following the total body fat (TBF) percentage?

Response options included:

1: Fixed percentages (independent from age) - Boys TBF% > 25% and girls TBF% > 30%

2: Fixed percentages (independent from age) - Boys TBF% > 25% and girls TBF% > 35%

3: Fixed percentages (independent from age) - Boys TBF% > 30% and girls TBF% > 32%

4: Fixed percentages (independent from age) - Boys TBF% > 30% and girls TBF% > 35%

5: Other fixed TBF percentages corresponding to country / region / ethnicity, independent from age

6: TBF percentiles by age and sex (i.e. > 95th percentile) by country / region / ethnicity.

7: Do not recommend the use of total body fat % for the reference standard.

8: Other

A total of 39 experts provided written or oral feedback, 25 attended the group meetings, and 23 of those 25 experts participated in the live polls in the meetings.

# Supplementary Figure 6

Poll 6: In my opinion, all measures of adiposity must be used with percentiles for age and sex, the use of fixed thresholds is not recommended.


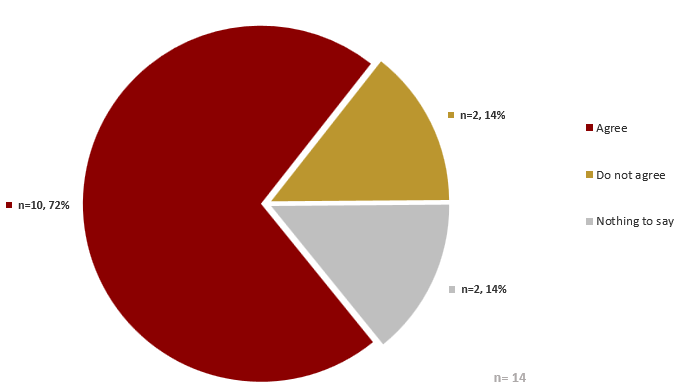


A total of 39 experts provided written or oral feedback, 25 attended the group meetings, and 23 of those 25 experts participated in the live polls in the meetings.

# Supplementary Figure 7

Poll 7: Rank the following measures of adiposity to be used with reference standards to assess body composition, from best (1) to worst (5).


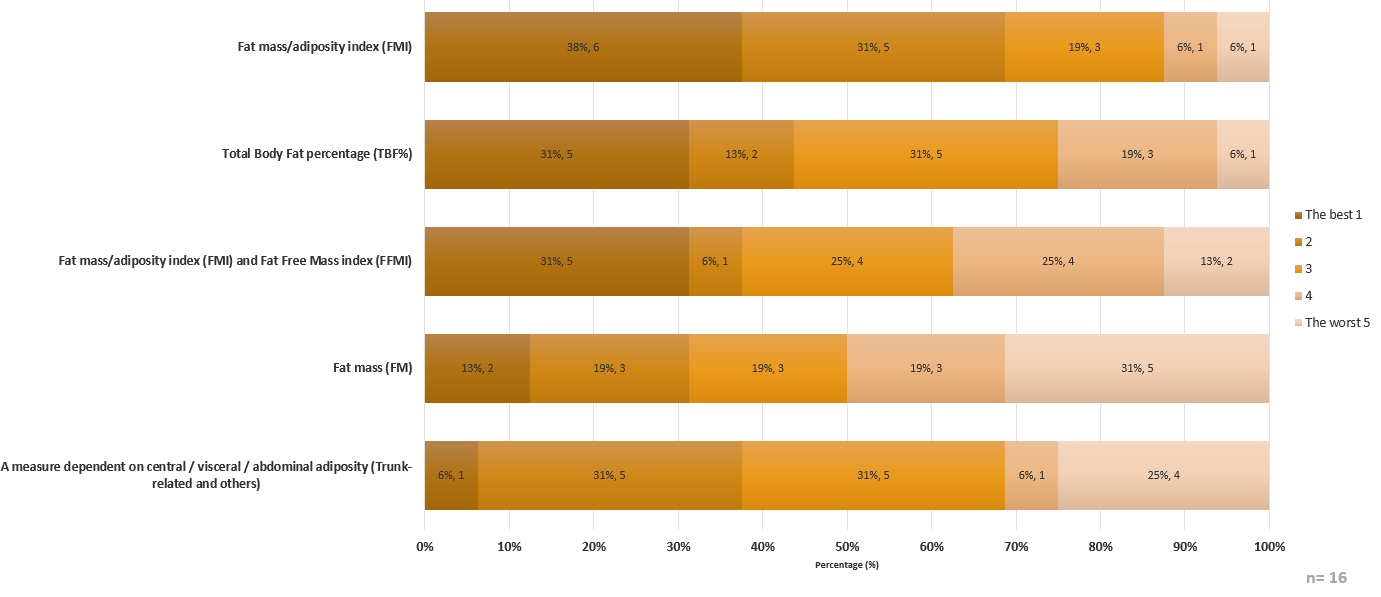


A total of 39 experts provided written or oral feedback, 25 attended the group meetings, and 23 of those 25 experts participated in the live polls in the meetings.

# Supplementary Figure 8

Poll 8: To accurately and reliably determine obesity in children and adolescents, it is necessary to:


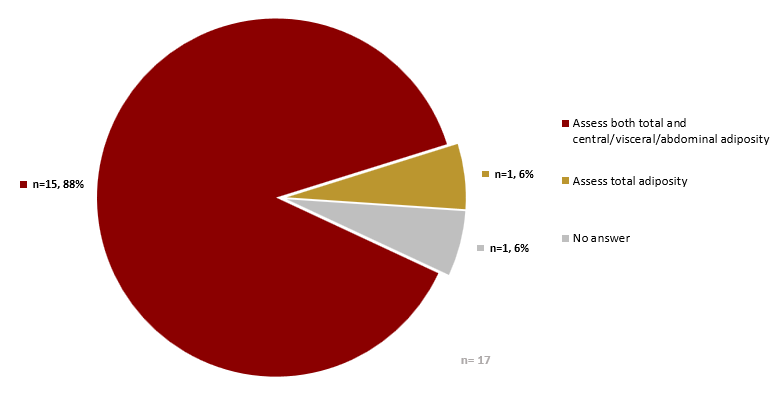


Response options included:

1: Assess total adiposity

2: Assess central/visceral/abdominal adiposity

3: Assess both total and central/visceral/abdominal adiposity

4: No answer

A total of 39 experts provided written or oral feedback, 25 attended the group meetings, and 23 of those 25 experts participated in the live polls in the meetings.
